# How Recent History Affects Perception: The Normative Approach and Its Heuristic Approximation

**DOI:** 10.1371/journal.pcbi.1002731

**Published:** 2012-10-25

**Authors:** Ofri Raviv, Merav Ahissar, Yonatan Loewenstein

**Affiliations:** 1 The Edmond & Lily Safra Center for Brain Sciences, Interdisciplinary Center for Neural Computation, Hebrew University, Jerusalem, Israel; 2 Departments of Psychology and Cognitive Sciences, Hebrew University, Jerusalem, Israel; 3 Departments of Neurobiology and Cognitive Sciences and the Center for the Study of Rationality, Hebrew University, Jerusalem, Israel; Brandeis, United States of America

## Abstract

There is accumulating evidence that prior knowledge about expectations plays an important role in perception. The Bayesian framework is the standard computational approach to explain how prior knowledge about the distribution of expected stimuli is incorporated with noisy observations in order to improve performance. However, it is unclear what information about the prior distribution is acquired by the perceptual system over short periods of time and how this information is utilized in the process of perceptual decision making. Here we address this question using a simple two-tone discrimination task. We find that the “contraction bias”, in which small magnitudes are overestimated and large magnitudes are underestimated, dominates the pattern of responses of human participants. This contraction bias is consistent with the Bayesian hypothesis in which the true prior information is available to the decision-maker. However, a trial-by-trial analysis of the pattern of responses reveals that the contribution of most recent trials to performance is overweighted compared with the predictions of a standard Bayesian model. Moreover, we study participants' performance in a-typical distributions of stimuli and demonstrate substantial deviations from the ideal Bayesian detector, suggesting that the brain utilizes a heuristic approximation of the Bayesian inference. We propose a biologically plausible model, in which decision in the two-tone discrimination task is based on a comparison between the second tone and an exponentially-decaying average of the first tone and past tones. We show that this model accounts for both the contraction bias and the deviations from the ideal Bayesian detector hypothesis. These findings demonstrate the power of Bayesian-like heuristics in the brain, as well as their limitations in their failure to fully adapt to novel environments.

## Author Summary

In this paper we study how history affects perception using an auditory delayed comparison task, in which human participants repeatedly compare the frequencies of two, temporally-separated pure tones. We demonstrate that the history of the experiment has a substantial effect on participants' performance: when both tones are high relative to past stimuli, people tend to report that the 2^nd^ tone was higher, and when they are relatively low, they tend to report that the 1^st^ tone was higher. Interestingly, only the most recent trials bias performance, which can be interpreted as if the participants assume that the statistics of stimuli in the experiment is highly volatile. Moreover, this bias persists even in settings, in which it is detrimental to performance. These results demonstrate the abilities, as well as limitations, of the cognitive system when incorporating expectations in perception.

## Introduction

Perception is a complex cognitive process, in which noisy signals are extracted from the environment and interpreted. It is generally believed that perceptual resolution is limited by internal noise that constrains our ability to differentiate physically similar stimuli. The magnitude of this internal noise is typically estimated using the 2-alternative forced choice (2AFC) paradigm, which was introduced to eliminate participants' perceptual and response biases [1], [2]. In this paradigm, a participant is presented with two temporally-separated stimuli that differ along a physical dimension and is instructed to compare them. The common assumption is that the probability of a correct response is determined by the physical difference between the two stimuli, relative to the level of internal noise. Performance is typically characterized by the threshold of discrimination, referred to as the Just Noticeable Difference (JND). Thus, the JND is a measure of the level of internal noise such that the higher the JND, the higher the inferred internal noise.

However, the idea that there is a one-to-one correspondence between the JND and the internal noise is inconsistent with theoretical considerations which postulate that participants' performance can be improved by taking into account expectations about the stimuli in the process of perception or decision-making. If the internal representation of a stimulus was uncertain, the prior expectations should bias the participant against unlikely stimuli. The larger the uncertainty, the larger the contribution of these prior expectations should be. The Bayesian theory of inference describes how expectations regarding the probability distribution of stimuli should be combined with the noisy representations of these stimuli in order to optimize performance [3].

In fact, expectations, formalized as prior distribution of stimuli used in the experiment, have been shown to bias participants' responses in a way that is consistent with the Bayesian framework (reviewed in [4]). In particular, responses in the 2AFC paradigm have been shown to be biased by prior expectations: when the magnitudes of the two stimuli are small relative to the distribution of stimuli used in the experiment, participants tend to respond that the 1^st^ stimulus was larger, whereas they tend to respond that the 2^nd^ stimulus was larger when the magnitudes of the two stimuli are relatively large [5]–[7]. In a previous study we have shown that this bias, known as the “contraction bias”, can be understood in the Bayesian framework: following the presentation of the two stimuli, the participant combines her noisy representations of the two stimuli with the prior distribution of the stimuli to form two posterior distributions. Rather than comparing the two noisy representations of the stimuli, the participant is assumed to compare the two posteriors in order to maximize the probability of a correct response. The contribution of the prior distribution to the two posteriors is not equal. The larger the level of noise in the representation of the stimulus, the larger is the contribution of the prior distribution to the posterior. The level of noise in the representation of the magnitude of the 1^st^ stimulus is larger than the level of noise in the representation of the magnitude of the 2^nd^ stimulus because of the noise associated with the encoding and maintenance of the 1^st^ stimulus in memory [8], [9]. As a result, the posterior distribution of the 1^st^ stimulus is biased more by the prior distribution than the posterior distribution of the 2^nd^ stimulus. If the prior distribution is unimodal, both posteriors are contracted towards the median of the prior distribution. Because the posterior of the 1^st^ stimulus is contracted more than the posterior of the 2^nd^ stimulus, participants' responses are biased towards overestimating the 1^st^ stimulus when it is relatively small and underestimating it when it is relatively large [7].

One limitation of the Bayesian model is that it relies heavily on the assumption that the prior distribution of stimuli is known to the observer. While this assumption may be plausible in very long experiments comprising a large number of trials (e.g. thousands in [10]) or in experiments utilizing natural tasks (e.g., reading, [11]), it is unclear how Bayesian inference can take place if participants have less experience in the task.

In this paper we study participants' pattern of responses in a 2AFC tone discrimination task in relatively short experiments consisting of tens of trials. We report a substantial contraction bias that persists even when it hampers performance due to a-typical statistics. We show that participants' pattern of behavior is consistent with an “implicit memory” model, in which the representation of previous stimuli is a single scalar that continuously updates with examples. Thus, this model can be viewed as a simple implementation of the Bayesian model that provides a better account of participants' perceptual decision making.

## Results

### The contraction bias

**Figure 1:**
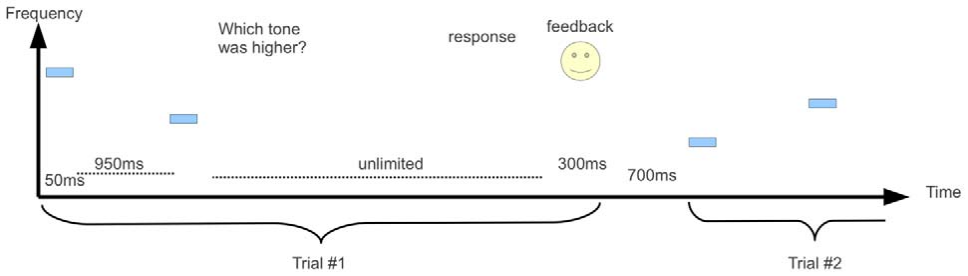
The experimental procedure. On each trial two 50 ms tones, separated by an interval of 950 ms, were played and the participant was asked to respond which of the 2 tones was higher by pressing a button. Immediately after the button press, visual feedback in the form of a smiling face for correct answers, and a sad face for incorrect answers was presented for 300 ms. The inter-trial-interval was 700 ms.; The two frequencies were drawn from a wide distribution and their ratio was determined by a staircase paradigm (see Materials and Methods).

We measured the performance of our participants in the random 2AFC paradigm (Materials and Methods, Fig. 1), in which subjects compared the frequencies of two sequentially presented tones drawn from a broad frequency range. Averaged across the population of participants, the JND was 13.6%±0.7% (SEM), which is higher than typically reported in the literature ([12], [13]). The relatively high value of the JND, which is likely to result from the lack of experience of the participants and the fact that no reference was used, is comparable with previous studies using the random frequency paradigm, with short stimuli and untrained participants [14], [15].

**Figure 2:**
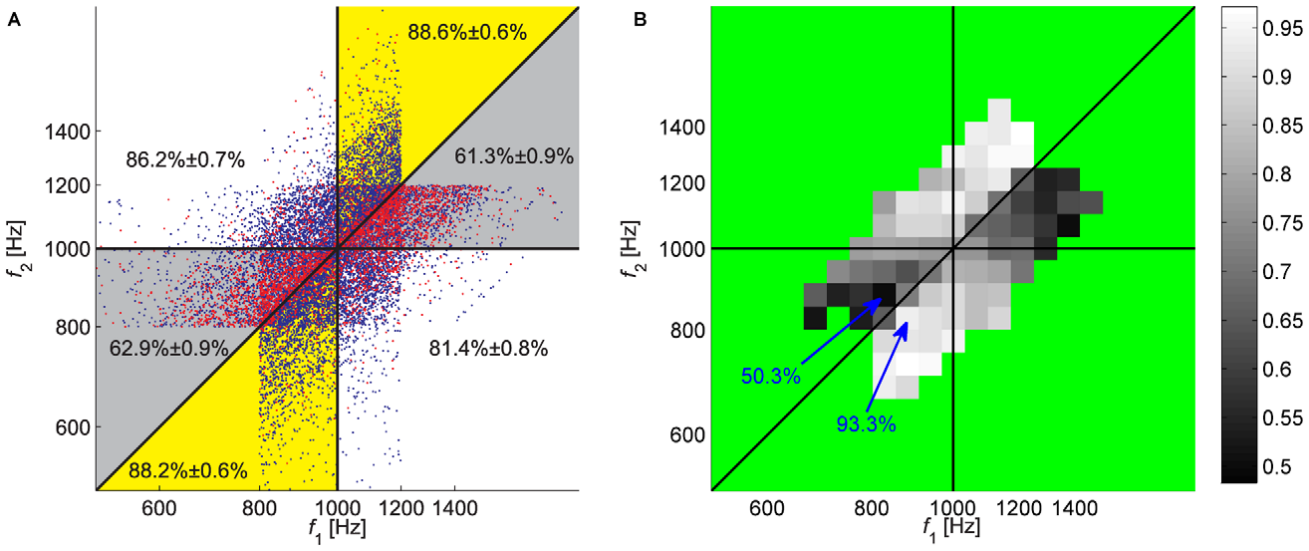
Performance of participants in Experiment 1. **A**. Pattern of responses. Each dot corresponds to one trial of one participant, where the axes denote the frequencies of the 2 tones in the trial: the abscissa is the frequency of the 1^st^ tone, 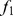, and the ordinate is the frequency of the 2^nd^ tone, 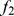, both on a logarithmic scale. The color of the dot denotes the outcome of the trial: correct responses are denoted by blue and incorrect responses by red. The vertical and horizontal lines correspond to the lines in which 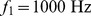 and 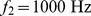, respectively. The diagonal line corresponds to the line in which 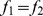. These lines partition the 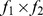 space into different regions, denoted using a different background color. The numbers in each region denote the fraction of correct responses in the region ± SEM. Note that the pattern of correct responses is not symmetrical with respect to the diagonal, as expected from a participant whose probability of success in the trial depends solely on the ratio of the two frequencies. **B**. A two-dimensional histogram of performance rate, computed by binning the data presented in **A** and computing the fraction of correct responses in each bin. Bins in which the number of trials was smaller than 50 were not analyzed and are colored green. Note in particular the 2 squares marked by arrows. Although they are of equal ‘objective’ difficulty (they are located at the same distance from the diagonal), performance differed substantially: in the square denoted by the upper arrow performance was at chance level (50.8% correct responses) whereas in the square denoted by the lower arrow it was 92.3%.

As predicted by the contraction bias, the JND did not capture the full pattern of participants' responses. This is depicted in Fig. 2A. The coordinates of each dot in Fig. 2A correspond to the frequencies of the 1^st^ and 2^nd^ tones in a trial, referred to as 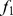 and 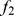. Blue and red dots denote trials, in which the participant's response was correct and incorrect, respectively. The closer the dots are to the diagonal, the smaller is the difference in the frequencies of the two tones. Therefore naively, one would expect that the probability of a trial to be incorrect (red dot) would be highest near the diagonal. Moreover, if the probability of a correct response as a function of 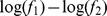 is symmetrical around 0, as implicitly assumed when measuring the JND, then the pattern of red and blue dots is expected to be symmetrical around the diagonal. In contrast, we found that the pattern of incorrect responses is highly non-symmetrical. Participants tended to err more when both frequencies were high and 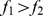 and when both frequencies were low and 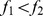. To quantify this asymmetry, we considered separately two regions: the Bias+ region corresponds to trials in two sections of this plane (yellow in Fig. 2A): in the first section are trials in which the frequencies of both stimuli are *above* the median (1000 Hz) and the frequency of the 1^st^ tone is *lower* than that of the 2^nd^ tone. In the second section are trials in which the frequencies of both stimuli are *below* the median frequency and the frequency of the 1^st^ tone is *higher* than that of the 2^nd^ tone. Similarly, The Bias− region (gray in Fig. 2A) corresponded to trials in which the frequencies of both stimuli are *above* the median (1000 Hz) and the frequency of the 1^st^ tone is *higher* than that of the 2^nd^ tone and trials in which the frequencies of both stimuli are *below* the median frequency and the frequency of the 1^st^ tone is *lower* than that of the 2^nd^ tone. Participants' rate of success differed greatly between the Bias+ and Bias− regions. Participants were typically successful when either the two tones were low (<1000 Hz) and the 2^nd^ tone was lower (lower left yellow region, 88.2%±0.5% correct responses, mean ± SEM) or when the two tones were high (>1000 Hz) and the 2^nd^ tone was higher (upper yellow region, 88.4%±0.6% correct responses). On the other hand, performance was relatively poor either when the two tones were low and the 1^st^ tone was lower (lower left gray region, 63.2%±0.8% correct responses) or when the two tones were high and the 1^st^ tone was higher (upper gray region, 61.8%±0.8% correct responses). These effects were highly significant in each of the two quadrants (*p*<10^−6^, Monte Carlo Permutation test). The differential level of proficiency in the yellow and gray regions indicates a substantial contraction bias, in line with that bias described in previous studies [6], [7]: when the frequency of the 1^st^ tone was relatively low, participants tended to overestimate it (leading to successful performance when the 1^st^ tone was higher). The opposite was true when the frequency of the 1^st^ tone was relatively high (leading to successful performance when the 1^st^ tone was lower). The differential level of proficiency in the yellow and gray regions is evident not only in the response pattern of the population of participants but also in the response pattern in individual blocks (Fig. S1A–C). Moreover, it was evident for all levels of proficiency in the task (Fig. S1D).

To further illustrate the contraction bias, we constructed a two-dimensional histogram of participants' performance by binning the 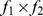 space of Fig. 2A and computing the fraction of correct responses in each bin (Fig. 2B, grayscale). The non-symmetrical distribution of the shades of gray of the squares around the diagonal reflects the contraction bias. Note in particular the two squares denoted by arrows. Despite the fact that they were of equal ‘objective’ difficulty (the absolute difference in frequencies was the same), the performance in the bottom right square region was almost perfect (92.2% correct responses; *n* = 324), whereas it was almost at chance level in the top left square region (50.8% correct responses; *n* = 323; *p*<10^−33^, Fisher's exact test). It should be noted that the bias in participants' response cannot be accounted for by a general preference in favor of one of the alternative answers, because the bias is opposite in the low and high frequencies.

The non-symmetrical performance around the diagonal (Fig. 2) is not captured by a single performance measure, the JND. This has motivated us to consider a measure of performance that captures some of this asymmetry. To that goal, we computed two separate JNDs for each participant (see Materials and Methods): one for the trials in the regions in which the contraction bias augments behavior (Bias+, yellow) and the other for the regions in which the contraction bias impairs behavior (Bias−, gray). These JNDs differed by more than 6 fold (the medians of JNDs across the population were 4.1%, and 27.0% for the Bias+ and Bias− regions, respectively; *p*<10^−5^, Monte Carlo Permutation test). In fact, as depicted in Fig. S2 in the Supporting Information section, a participant's proficiency on a trial depended more on the contraction bias (i.e. Bias+ versus Bias− regions) than on the participant's overall proficiency (overall low versus high JND). These results demonstrate the substantial contribution of this bias to behavior.

### Recency effect and the prior distribution

In a previous study we have shown that the contraction bias in a visual discrimination task is consistent with a model of an ideal detector that utilizes Bayes' rule to incorporate the prior distribution with the sensed stimuli in order to optimize performance [7]. In agreement with that study, such a Bayesian model, with 2 free parameters that correspond to the noise in the internal representation of each of the two stimuli, can qualitatively account for the observed contraction in the two-tone discrimination task (see Fig. S3 in the Supporting Information section).

However, it should be noted that the Bayesian model relies on the assumption that the prior distribution of stimuli is known to the observer, which seems unreasonable in our experiment, which consisted of merely tens of trials. Therefore, it is not clear how the history of trials experienced by the participants in the experiment contributes to the bias. To address this question, we considered the contribution of individual trials to the bias. Because the statistics of stimuli in our experiment are stationary, all past trials are equally informative about the prior distribution. Therefore, normative considerations that incorporate an assumption of stationarity imply that the effect of past trials on participants' choices will be independent of the number of trials elapsed between these trials and the choice. By contrast, previous studies have reported that participants' responses are influenced to a greater degree by recent stimuli, which is known as the recency effect [16]–[21]. In addition, the activity of neurons in the primary auditory cortex has been shown to contain information about both current and previous stimuli [22]. To test for recency in our dataset, we fitted a linear non-linear model that relates the response in each trial to a linear combination of present and past stimuli according to the following equation:
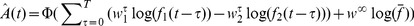
(1)where 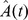 is the probability that the model would report that the frequency of the 1^st^ tone was higher than that of the 2^nd^ tone in trial 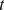; 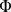 is the normal cumulative distribution function such that 
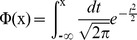
; 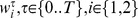 and 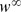 are parameters, 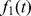 and 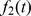 are the frequencies of the 1^st^ and 2^nd^ tone, respectively, in trial 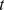 and 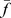 is the geometric mean of the frequencies of all stimuli in the experiment until trial 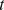.

To gain insights into the behavior of the model (Eq. (1)) we consider the simple case in which 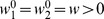 and 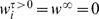. In this case, Eq. (1) becomes 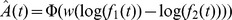, which corresponds to a model participant that is indifferent to the history of the experiment and its choices depend solely on the ratio of the frequencies of the two tones and the internal noise. The value of 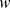 denotes the level of internal noise of the model participant. If 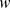 is very small, 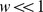 then independently of the frequencies of the stimuli, 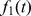 and 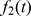, 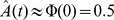, and the model participant responds at random. In contrast, if 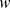 is very large, 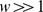 then 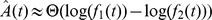 where 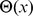 is the Heaviside step function such that 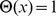 for 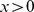 and 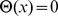 for 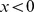. In other words, if 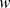 is very large the model participant always answers correctly. The larger the value of 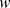, the smaller the JND of the model participant. The values of the parameters 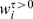 determine the contribution of past stimuli to perception, where the value of 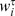 determines the contribution of the 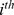 stimulus presented 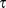 trials ago and the value of 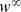 determines the contribution of the average frequency of past stimuli to perception. If all past stimuli contribute equally to perception, as expected from normative participants who assume that the distribution of stimuli is stationary then we expect 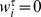 and 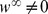. In contrast, if the participant assumes that the statistics of the experiment is non-stationary then we expect the most recent trials to have a stronger effect on behavior, resulting in 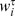 whose magnitude decreases as the value of 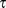 increases.

**Figure 3:**
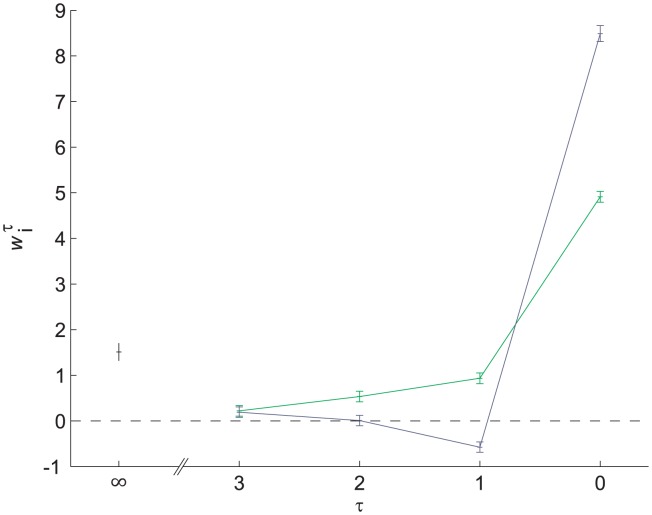
Recency effect. To estimate the effect of stimuli administered in previous trials on decision in a trial, we fitted a linear non-linear model that relates the outcome of each trial to a linear combination of present and past stimuli (Eq. 1). The parameters that minimize the square error between the prediction of the model and participants' responses are presented. Green - 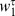, Dark blue - 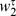, Black - 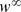. Error bars are 68% confidence intervals (equivalent to one standard deviation in a normal distribution) and we assumed that 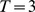, which means that the model had 9 free parameters (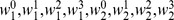 and 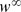), and was fitted using 16,380 trials (65 trials in 252 blocks).

Assuming that 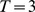, we analyzed the sequence of frequencies and decisions of our participants. We found the values of the parameters 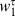 (Fig. 3, green), 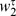 (dark blue) and 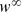 (black) that minimize the mean square error (MSE), the mean square distance of the vector of probabilities, 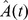 from the vector of choices, 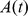 such that 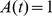 if the participant responded that the frequency of the 1^st^ tone was higher than the frequency of the 2^nd^ tone in trial 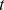 and 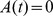 otherwise. Note that the values of 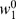 and 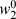 in Fig. 3 are larger than the values of all other coefficients, 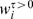. This result reflects the simple fact that the tones presented in a trial influence the decision in that trial more than tones presented in previous trials. The recency effect is manifested in the non-zero coefficients of 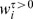 (see Materials and Methods). As depicted in Fig. 3, the contribution of past trials to choice diminishes within several trials. This result is consistent with other findings of rapid perceptual learning [23], [24] (but see also [25]) and demonstrates that at least some aspects of the prior distribution are estimated using a small number of the most recent trials. It should also be noted that the contribution of past stimuli to decision is dominated by past values of 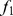 and not past values of 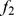 (Fig. 3. See also Materials and Methods).

### The implicit-memory model

The recency effect described in the previous section is difficult to reconcile with a Bayesian inference model that takes into account the stationary statistics of the experiment. This finding has motivated us to consider the possibility that the contraction bias described in Fig. 2 emerges from simpler cognitive processes that do not require an explicit representation of the prior distribution. In this section we present a simple model that accounts for the contraction bias and the recency effect, which does not explicitly keep track of the prior distribution of stimuli presented in the experiment.

In our model, the memory trace of past stimuli is a single scalar 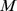 (rather than the full prior distribution). In response to the presentation of 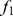, the participant updates the value of 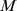 such that 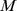 is a linear combination of the past value of 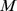 with the present stimulus, corrupted by sensory and encoding noise. Formally, the value of 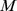 in trial 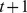, is given by
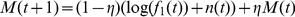
(2)where 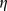 is the weight given to the memory and 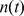 is the noise associated with the encoding of 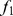. We assume that this noise is Gaussian with variance 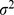 and is uncorrelated across trials: 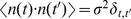, where 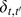 is the Kronecker delta function, 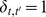 if 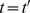 and 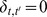 if 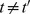.

A decision in a trial in this model depends on the relative values of 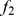 and 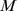. If 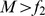, the model responds that “
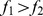
”. Otherwise it responds that “
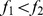
”. In this model we assume that the noise is restricted to the representation of 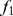. The reason for ignoring noise in the representation of 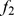 is that noise in 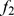 is mathematically equivalent to a larger magnitude noise in 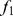 when considering decision in a given trial.

It is easy to show that in this model, 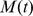 is an exponentially weighted sum of the current and past stimuli and their respective encoding noises:
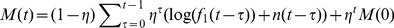
(3)Note that in this model past values of 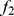 do not contribute to behavior. This reflects the dominance of past values of 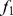 in Fig. 3 (see also Materials and Methods). It should also be noted that in this model, the contribution of past stimuli to decision (which plays the role of the prior distribution in the Bayesian model) is encoded using the same variable as the encoding of 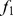. Therefore, the model does not require any form of separate representation of the long term memory of past trials.

**Figure 4:**
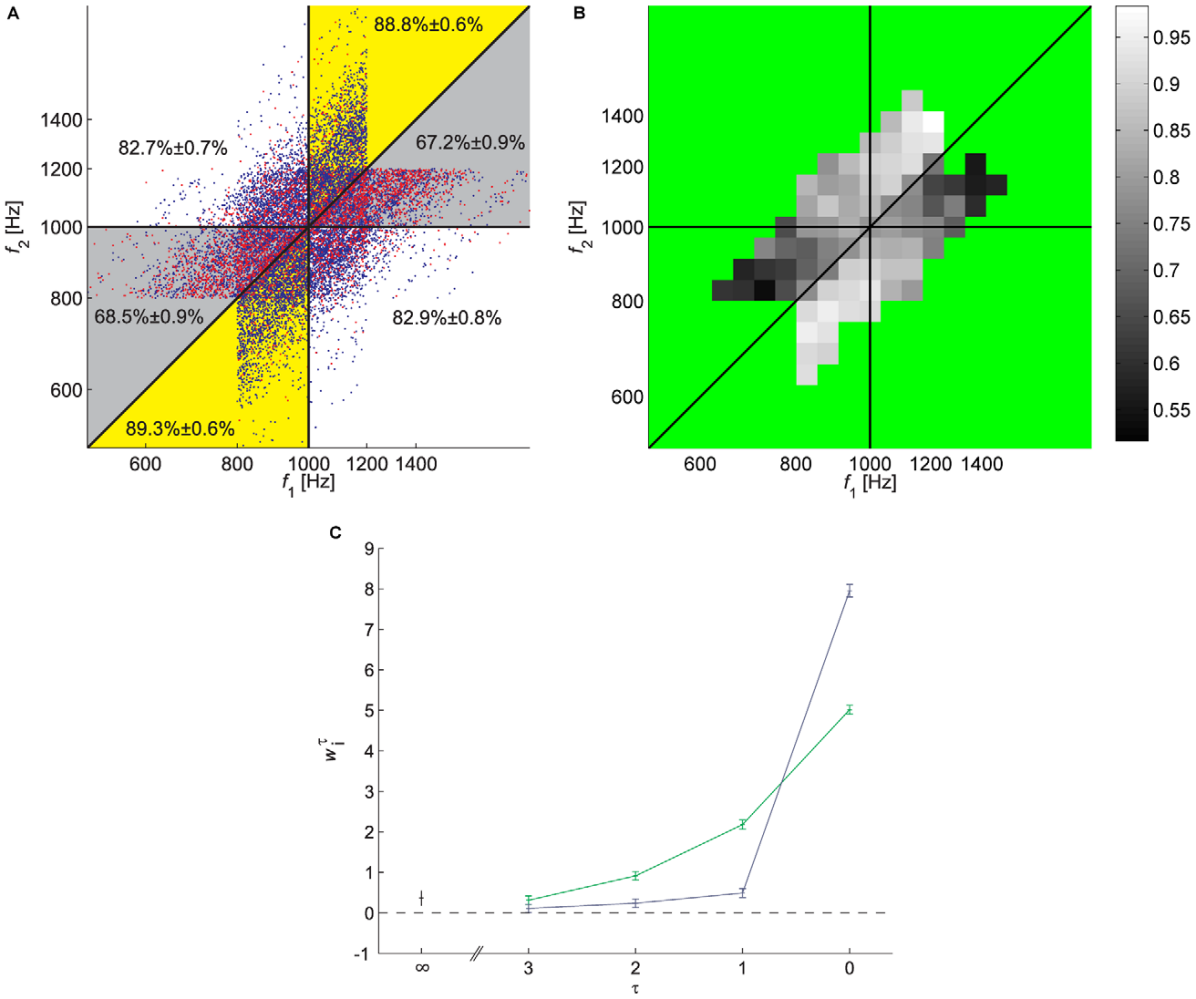
The implicit memory model. The parameters of the implicit memory model, the standard deviation of the noise, 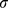 and the memory weight, 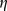 were estimated for each of our experimental blocks to minimize the square error between the model and the observed behavior. These parameters were used to simulate the behavior of an implicit-memory participant in that block. The results of the simulation are presented in **A** and **B**, (same presentation as in Fig. **2A** and **2B**, respectively). Note the similarity between Figs. **4A** and **2A** and between Figs. **4B** and **2B**, indicating that the implicit-memory model can account for the contraction bias. **C**, Estimation of the recency effect in the implicit memory model. Same analysis as in Fig. **3**.

The implicit-memory model is characterized by two parameters that denote the level of noise, 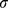 and the extent to which the history of the experiment affects perception, 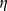. Fig. 4 depicts the results of a simulation of a population of implicit memory models, each with the parameters 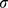 and 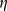 best fitting a single block in our dataset (see Materials and Methods). As shown in Figs. 4A and 4B, the model results in a contraction bias, which is comparable to the experimentally observed (compare Figs. 4A and 4B to Figs 2A and 2B, respectively). A quantitative analysis reveals that the goodness-of-fit of the Implicit memory model is comparable to that of the Bayesian model (Fig. S4). However, in contrast to be Bayesian model that assumes a constant prior, the contribution of very recent trials to performance (Eq. (1)) in the Implicit memory model is similar to that of our participants (compare Fig. 4C to Fig. 3).

### The rigidity of the contraction bias

The contraction bias in Fig. 2 can be justified using optimality considerations, in which prior knowledge is incorporated with the observations in order to maximize performance (Fig. S3). Would contraction bias persist in an experiment in which it impairs performance due to the dependencies between the frequency distribution of the two tones?

**Figure 5:**
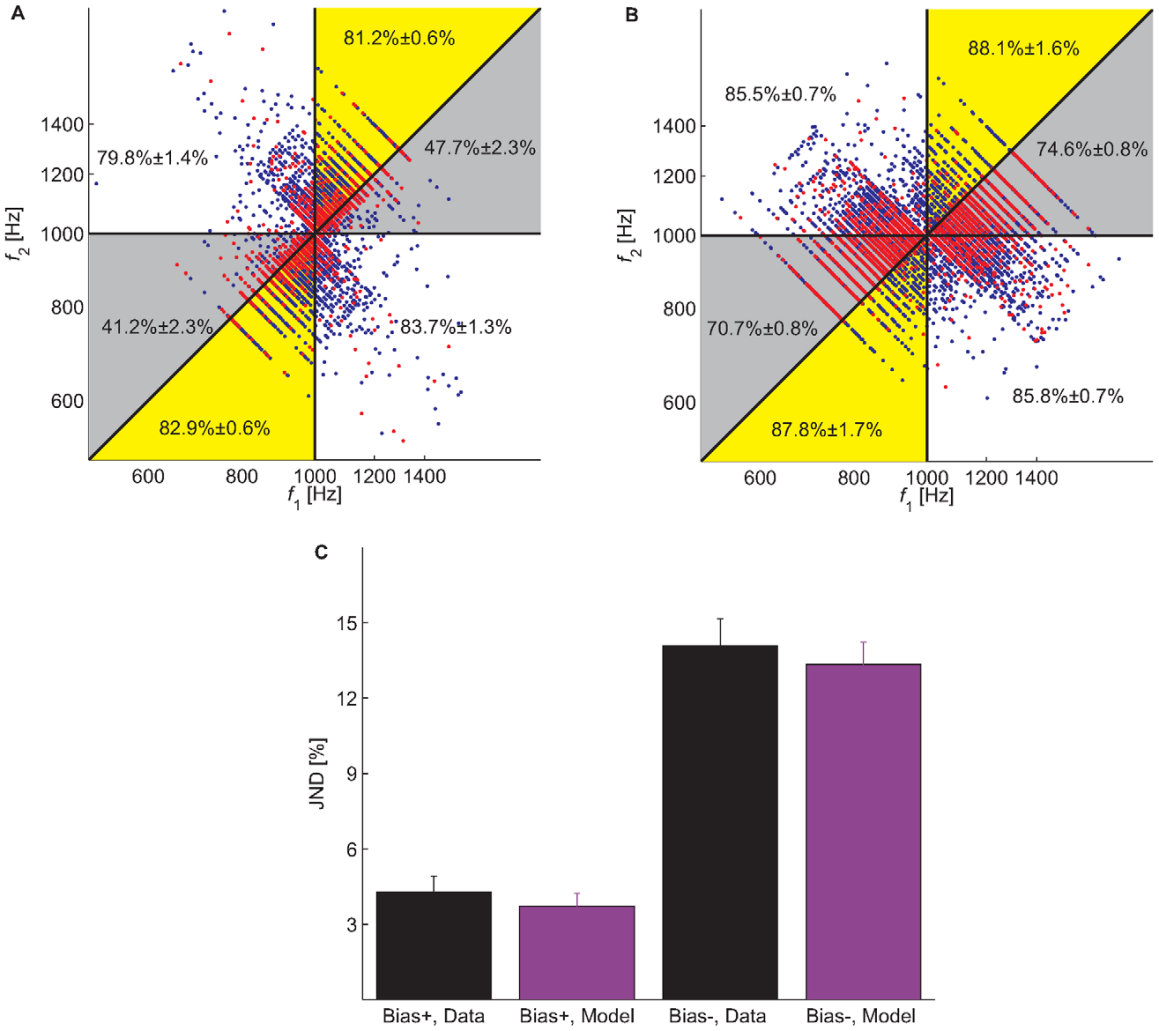
Results of Experiment 2. **A**, Pattern of responses in the Bias+ condition, in which the fraction of trials in the Bias+ region (yellow) is larger than the fraction of trials in the Bias− region (gray). **B**, Pattern of responses in the Bias− condition, which oversamples the Bias− region. Same presentation as in Fig. **2A**. **C**, Experimental (black) and Implicit Memory Model simulation (purple) Mean ± SEM JND in the Bias+ (left) and Bias− (right) conditions. In the simulations, the parameters of each block were estimated in the Bias+ condition and were used to simulate the implicit memory model in both the Bias+ and in the Bias− conditions.

In order to address this question, we conducted a second experiment (Experiment 2 in the Materials and Methods), in which we manipulated the correlations between the frequencies of the two tones such that in some blocks the contraction bias is beneficial to performance whereas in others it is detrimental. Contraction bias is beneficial in the Bias+ region (yellow in Figs. 2A and 4A) and is detrimental in the Bias− region (gray in Figs. 2A and 4A). Therefore, in this experiment we manipulated the fraction of trials in the Bias+ and Bias− regions in different blocks. In one condition, the two tones were chosen such that the 2^nd^ tone was typically higher than the 1^st^ when the two frequencies were relatively high, and the 2^nd^ tone was typically lower than the 1^st^ when the two frequencies were relatively low. We refer to this condition as the ‘Bias+ condition’, because there were many more trials in the Bias+ region than in the Bias− region (11,233 vs. 1172). In the other condition, the two tones were chosen such that the 1^st^ tone was typically higher than the 2^nd^ when the frequencies of the two tones were relatively high and the 1^st^ tone was typically lower than the 2^nd^ when the frequencies of the two tones were relatively low. This ‘Bias− condition‘ was comprised of substantially more trials in the Bias− region than in the Bias+ region (8111 vs. 952). Figs. 5A and 5B depict the distribution of trials and correct and incorrect responses in the Bias+ and Bias− conditions, respectively. Similar to the pattern of responses in the first experiment (Fig. 2A), participants were more likely to be correct in the Bias+ regions, compared to the Bias− regions. This was true both for the Bias+ condition (82.0%±0.4% correct responses vs. 44.5%±1.6% correct responses, p<10^−126^ Fisher exact test) and the Bias− condition (88.0%±1.2% correct responses vs. 72.6%±0.6% correct responses, *p*<10^−21^ Fisher exact test). The JNDs were significantly different in the two conditions: the mean JND in the Bias+ condition was only 4.3%±0.6%, compared to 14.1%±1.1% in Bias− condition (Fig. 5C, black, *p*<10^−25^, Wilcoxon rank sum test).

In the framework of the Bayesian model, the difference in proficiency between the two conditions is surprising because given the joint distribution, the detection problem in the two conditions is symmetric. However, our results indicate that our participants did not utilize these probabilities when making a decision about the relative frequencies in this task.

To test the ability of the implicit-memory model to account for the results of the second experiment, we fitted the parameters of the model (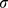 and 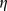) to the experimental data of the Bias+ condition. We then simulated each of the model participants in both the Bias+ and Bias− conditions. The resulting JNDs (mean ± SEM 3.7%±0.5% and 13.3%±0.9% for the Bias+ and Bias− conditions, respectively, purple in Fig. 5C) are not statistically different from to the experimentally measured JNDs (4.3%±0.6% and 14.1%±1.6%; *p* = 0.78 and *p* = 0.85, respectively, Wilcoxon rank sum test), suggesting that the participants did not utilize the differential statistics of the two tones in the two conditions. For example, they did not decrease the weights of recent trials even when their performance was consequently hampered. In fact, adapting to the Bias− condition simply by setting the weight of the history-dependence parameter 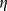 to 0 (effectively eliminating the contribution of past stimuli to decision in the model) would have improved their performance. To demonstrate this, we simulated the model participants in the Bias− condition while assuming that 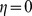. The resultant JND was only 9.1%±0.7%, lower than the JND of the model participants when assuming the history-dependence parameter 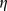 measured in the Bias+ condition.

## Discussion

In this work we showed that the contraction bias is a dominant determinant of participants' behavior in a 2AFC tone frequency discrimination task. Some aspects of this bias are consistent with the behavior of an ideal detector that utilizes the prior distribution to maximize performance. However, a substantial recency effect combined with a failure of the participants to utilize the joint distribution of the stimuli implies that this Bayesian-like computation is approximated using a much simpler algorithm, in which the prior distribution is not fully represented.

What information does the cognitive system store about the prior distribution? The full Bayesian model represents one extreme approach, in which it is assumed that the participant has full information about the joint distribution of the two stimuli. The standard way in which signal detection theory is applied to psychophysics represents the other extreme, in which the participant does not have (or does not utilize) any prior information about the identity of the stimuli (but only about the probability of each response being correct [1]). The contraction bias in Fig. 2 demonstrates that participants have some information about the marginal probabilities. However, the strong recency effect (Fig. 3) indicates that this marginal probability is constantly updated using a small number of most recent observations, even in stationary environments. In a normative framework, the recency effect, observed previously in various tasks [26], [27], implies that participants believe that the environment is highly volatile and as a result only the very recent history is informative about future stimuli.

The results of experiment 2 (Fig. 5) indicate that participants are either unable to compute the joint distribution or unable to utilize it, at least within a single experimental block of 80 trials. The implicit memory model can be viewed as a minimal modification of the standard approach of applying signal detection theory to perception in the direction of the full Bayesian model. Here, participants represent the prior distribution of the stimuli with a single scalar, which is an estimate of the mean of the marginal of the prior distribution. Nevertheless this implicit model captures many aspects of the behavioral results. Further studies are needed to determine whether, and to what extent other moments of the prior distributions are learned and utilized in the 2AFC discrimination task, especially under longer exposure to distribution statistics.

Several studies have shown that the magnitude of the contribution of the prior distribution to perception on a given trial depends on the level of internal noise [10], [28]. In particular in the framework of the 2AFC task, increasing the delay between the 1^st^ and 2^nd^ stimuli [29], [30] or introducing a distracting task between them [7] enhances the contraction bias. These results are consistent with the Bayesian approach. How can these results be accounted for in the framework of the implicit memory model? One possibility is to assume that the relative contribution of the prior in the simplified online rule of Eq. (2) is affected by perceptual noise. However, it should be noted that at least in one case, the level of noise was determined *after* the encoding of the 1^st^ stimulus [7]. The dependence of 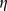 on the level of noise can be accounted for in the framework of the implicit memory model if we assume that the computation of 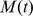, which incorporates the prior knowledge with the response to the 1^st^ stimulus, is carried out simultaneously by several neurons, or populations of neurons, which are characterized by different values of 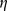 [22], [31], [32]. At the time of the decision, the magnitude of the noise determines which populations of neurons will be the most informative with respect to the 1^st^ stimulus. If the level of noise is high, the populations of neurons that are more affected by past trials (for whom the value of 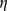 is large) will dominate perception, resulting in a substantial contraction bias. Otherwise the populations that are less affected by past trials will dominate perception, resulting in a small contraction bias.

Almost 40 years ago, Tversky and Kahneman characterized irrational decision making and reasoning and concluded that “people rely on a limited number of heuristic principles which reduce the complex tasks … to simpler judgmental operations. In general, these heuristics are quite useful, but sometimes they lead to severe and systematic errors” [33]. Our study extends these results to the domain of implicit perceptual judgments.

## Materials and Methods

### Ethics Statement 

The research was approved by the department ethics committee, and all participants signed consent forms.

### Experiment 1

150 participants (mean age 24±3.1 years) engaged in a 2AFC high/low pure tone frequency discrimination task, after signing consent forms. 18 participants were excluded due to poor performance on a hearing test or because they did not complete the full schedule. Each participant performed 2 blocks of 80 trials. Each trial consisted of two 50 ms pure tones, with 10 ms linear rise time, and 10 ms linear fall time, separated by 950 ms. Immediately after the 2^nd^ stimulus was played, the text ‘Which tone was higher?’ appeared on screen, and the participant responded by clicking one of two on-screen buttons using a computer mouse, with no time constraint. Visual feedback of a smiling face or a sad face was presented for 300 ms after correct and incorrect responses, respectively. After a pause of 700 ms the next trial began (Fig. 1). All stimuli were presented binaurally through Sennheiser HD-265 linear headphones using a TDT System III signal generator (Tucker Davis Technologies) controlled by in-house software in a sound attenuated room in the laboratory. Tone intensity was 65 dB SPL. Both the 1^st^ and the 2^nd^ frequencies in each trial were drawn from a wide distribution according to the following procedure: a frequency 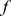 was drawn from a uniform distribution between 800 Hz and 1200 Hz. Another frequency, either 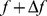 or 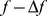 was drawn with a probability 0.5, where 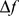 was controlled by an adaptive 3-down 1-up staircase, in which the initial difference between the stimuli in each block was 20% and was bounded from below by 0.1%. The step size decreased every four reversals, from 4.5% to 2% to 1% to 0.5% to 0.1%. One of the two frequencies was randomly selected as 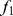 and the other frequency was selected as 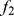. This schedule is expected to converge to a 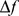 for which the participant answers correctly in 79.4% of the trials ([34]; Fig. 2A, dots). Blocks that did not converge to at least 65% correct responses in the last 40 trials were excluded from the analysis (12 of 264 blocks). The JND was calculated as the average difference between the stimuli frequencies in the last 6 reversals. As a result of the adaptive staircase schedule, the ratios between the frequencies of the two stimuli tended to decrease in the first trials of the block. On average, after 15 trials this ratio stabilized and therefore the first 15 trials of each block were excluded from the analysis.

### Estimating the JND in Bias+ and Bias− regions

To estimate the JND in a Bias+ or Bias− region of a block, we fitted a cumulative normal distribution function psychometric curve that relates the response in each trial 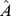 to the difference in the logarithm of the 1^st^ and 2^nd^ frequencies: 
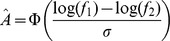
 where 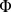 is the normal cumulative distribution function, such that 
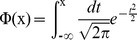
. The value of the parameter 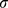 was chosen as to minimize the square difference between the vector predictions 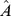 and the vector of choices 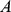 such that 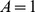 on trials in which the participant responded “
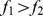
” and 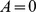 otherwise. Assuming that the cumulative normal distribution function reflects the probability of responding “
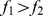
”, the corresponding value of the JND is the difference in the natural logarithms of 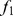 and 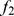 such that the probability of a correct response is the asymptotic performance level in our staircase paradigm, 0.794. Therefore, 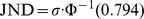.

### Statistical methods

To test for differences in performance between different regions, we used a Monte Carlo permutation test in which the identities of 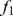 and 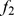 in a trial were randomly shuffled. We used 10^6^ permutations, and in all cases the experimentally observed differences were larger than the differences observed in all permutations, resulting in *p*<10^−6^.

To test for differences in the JNDs between different regions, we used a Monte Carlo permutation test in which the identities of 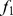 and 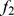 in a trial were randomly shuffled. We estimated the JND of these simulated results using the same process as described for the data, and estimated the median JND+ and median JND- for the whole population. We used 10^5^ permutations and the experimentally observed difference was larger than the difference observed in all permutations, resulting in *p*<10^−5^.

In order to verify the contribution of the parameters 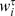 for 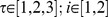 to the linear-non-linear model (Eq. 1), we compared several models using a cross validation test: the parameters of the different models were estimated using all blocks but one, and these parameters were used in order to compute the MSE for that block. The MSE of the model was computed by repeating this procedure for all blocks in the experiment and averaging the resultant MSE.

We considered three models: (1) a naïve model with no history dependence: 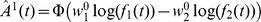; (2) a model with a global history term, 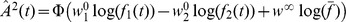; (3) the full model with an explicit history dependence of three previous trials, and a global term, 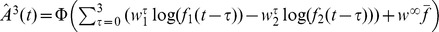. The resultant MSEs are 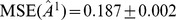; 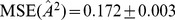; 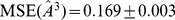. We found that 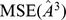 is significantly smaller than 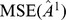 and 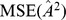 (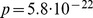 and 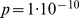 respectively, Wilcoxon signed rank test).

In order to verify that the contribution of past trials is dominated by values of 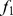, we compared two additional models, using the same analysis as above: (4) a model in which the recent history is represented by 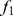 only: 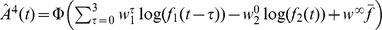; (5) a model in which the recent history is represented by 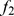 only: 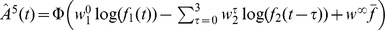. The resultant MSEs are 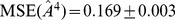 and 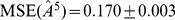. While 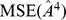 is not statistically different from 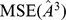 (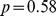), 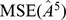 is significantly higher (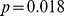) indicating that the model with only coefficients corresponding to the contribution of 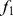 is as predictive as the full model.

### Experiment 2

Experiment 2 was similar to experiment 1, except for the joint distribution of 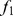 and 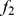: in each trial, a frequency 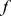 was chosen such that the natural logarithm of 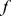, measured in Hz, was drawn from a normal distribution with mean 6.908 (corresponding to 1000 Hz), and standard deviation 0.115. In all trials, the mean of 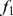 and 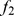 (in the logarithmic domain) was 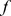. Another frequency, either 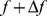 or 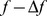 (in the logarithmic domain) was drawn with a probability 0.5, where 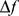 was controlled by an adaptive 3-down 1-up staircase schedule. In contrast to Experiment 1, the *order* of frequencies was *biased* and depended on 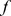. In trials in which 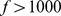, 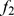 was chosen to be larger than 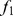 with a probability 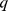. In contrast, in trials in which 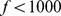, 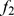 was chosen to be larger than 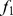 with a probability 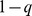. We studied two conditions: in one condition, which we refer to as “Bias+”, 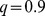. In the second condition, referred to as “Bias−”, 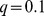. 60 participants (mean age 23.8±3.3 years) that did not participate in experiment 1 performed 6 interleaved blocks of Bias+ and Bias− conditions, with the order counterbalanced across participants. Similar to experiment 1, each block consisted of 80 trials.

### Fitting implicit memory model parameters

Rewriting Eq. (3), 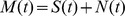 where 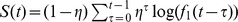 is a “signal” term that depends on previous trials and 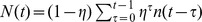 is a “noise” term. The probability of responding “
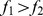
” response is thus given by 
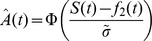
, where 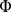 is the normal cumulative distribution function, and 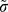 is the standard deviation of 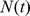. Because we excluded the first 15 trials from our analysis, we assumed that 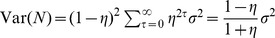. We fitted the pair 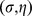 to the remaining 65 trials of each block to minimize the square error between the predictions of the model 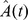 and the actual responses, 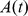.

## Supporting Information

Figure S1 **Performance of participants in Experiment 1 as a function of the JND.** **A–C**, Three representative blocks demonstrating contraction bias in single blocks. The three blocks correspond to the 15^th^, 50^th^ and 85^th^ percentile of the JNDs, respectively, Same presentation as in Fig. **2A**. The fraction in each region corresponds to the number of correct responses in that region divided by the total number of trials there. **D**, Contraction bias as a function of the JND. The blocks were divided to 10 groups of approximately equal number of blocks (25–26 blocks). For each group, we report the fraction of correct responses ± SEM in the Bias+ (yellow) and Bias− (gray) regions. The horizontal lines correspond to the ranges of JNDs in each group.
(EPS)

Figure S2 **Cumulative distribution of JNDs.** Blue and red denote the cumulative distribution of JNDs of good and poor performers, respectively, as measured in the Bias+ (solid lines), and Bias− regions (dashed lines). Good/poor performers are defined as participants whose overall JND, measured for all trials, was below/above the median JND. As expected, good performers were better than poor performers even when considering the Bias+ and Bias− regions separately (solid blue line is above solid red line and dashed blue line is above dashed red line). As predicted from the contraction bias, performance in the Bias+ region was higher than in the Bias− region (solid blue line is above dashed blue line and solid red line is above dashed red line). Note that poor performers in the Bias+ regions (solid red line) performed better than good performers in the Bias− regions (dashed blue line). This indicates that the region is more informative about performance in a trial than whether the participant belongs to the group of good or poor performers.
(EPS)

Figure S3
**The Bayesian model.** The parameters of the Bayesian model, the standard deviations of the noise in the representation of the two stimuli, 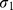 and 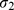 were estimated for each of our experimental blocks to minimize the square error between the model and the observed behavior (see ‘Fitting the Bayesian model parameters’ in the Supporting Information section). These parameters were used to simulate the behavior of a Bayesian-model participant in that block. The results of the simulation of the Bayesian models in all blocks are presented in **A** and **B**. In the same presentation as in Figs. **2A and 2B**. Note the similarity between Fig. **S3A** and Fig. **2A** and between Fig. **S3B** and Fig. **2B**, demonstrating that the Bayesian model can account for the contraction bias observed in the experiment.(TIF)

Figure S4 **Goodness of fit of the Bayesian and Implicit memory models in Experiment 1.** Each dot corresponds to the MSE of the Bayesian model as a function of the MSE of the Implicit memory model in a single block. In 55% (138/252) of the blocks the Bayesian model outperformed the Implicit Memory model but the difference is not statistically significant (*p* = 0.07, Wicoxson signed rank test).
(EPS)

Text S1 **Fitting the Bayesian model parameters.** Assumptions and implementation details for the Bayesian model fitted to the data.
(PDF)
