# Supplementary figures and images for: How Recent History Affects Perception: The Normative Approach and Its Heuristic Approximation
Source: PLoS Comput Biol. 2012 Oct 25;8(10):e1002731. doi: 10.1371/journal.pcbi.1002731 (PMC3486920; doi:10.1371/journal.pcbi.1002731)

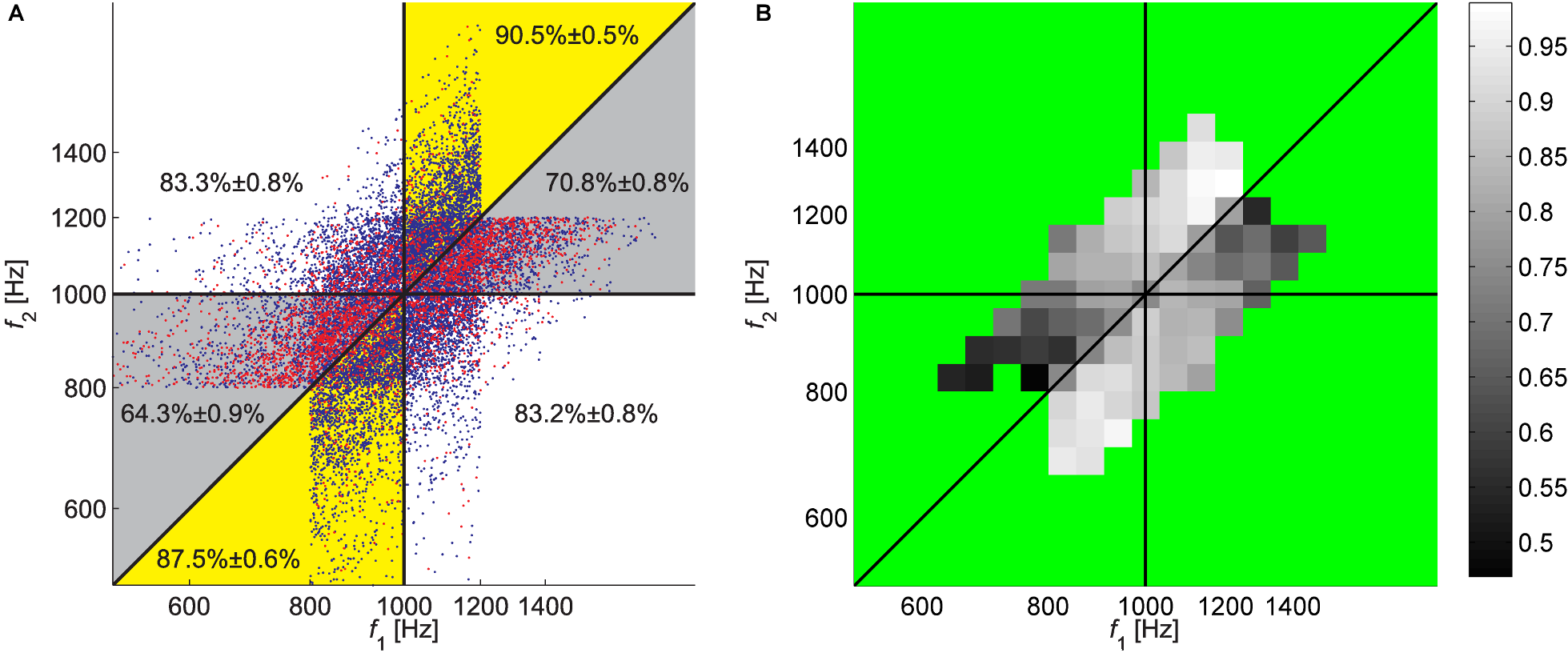

Supplement: Figure S3 — The Bayesian model. The parameters of the Bayesian model, the standard deviations of the noise in the representation of the two stimuli, and were estimated for each of our experimental blocks to minimize the square error between the model and the observed behavior (see ‘Fitting the Bayesian model parameters’ in the Supporting Information section). These parameters were used to simulate the behavior of a Bayesian-model participant in that block. The results of the simulation of the Bayesian models in all blocks are presented in A and B. In the same presentation as in Figs. 2A and 2B . Note the similarity between Fig. S3A and Fig. 2A and between Fig. S3B and Fig. 2B , demonstrating that the Bayesian model can account for the contraction bias observed in the experiment. (TIF) [file pcbi.1002731.s003.tif]
