# Supplementary material for: How Recent History Affects Perception: The Normative Approach and Its Heuristic Approximation
Source: PLoS Comput Biol. 2012 Oct 25;8(10):e1002731. doi: 10.1371/journal.pcbi.1002731 (PMC3486920; doi:10.1371/journal.pcbi.1002731)
Supplement: Text S1 — Fitting the Bayesian model parameters. Assumptions and implementation details for the Bayesian model fitted to the data. (PDF) [file pcbi.1002731.s005.pdf]

Fitting the Bayesian model parameters

The Bayesian-inference model used to fit the participants' responses in a block drew on the following three assumptions: (1) The distributions of f_1 and f_2 are Gaussian (in the logarithmic scale) where the means and variances of the two distributions are the empirical means and variances of that block. (2) Each observation of a frequency f_i is corrupted by a zero mean Gaussian noise (in the logarithmic scale). (3) The participant uses Bayesian inference to compute the posterior distributions of f_1 and f_2 given the noisy observations and the prior distribution. The participant then chooses his / her responses according to these posteriors to maximize the probability of a correct response. Thus, the probability that a model participant would respond that the 1st frequency is higher than the 2nd frequency in a trial in which the frequencies f_1 and f_2 are played, $\Pr(\text{"}f_1 > f_2\text{"} \mid f_1, f_2)$ is given by

$$\Pr(\text{"}f_1 > f_2\text{"} \mid f_1, f_2) = \int_{-\infty}^{\infty}\int_{-\infty}^{\infty} \Pr(r_1 \mid f_1)\Pr(r_2 \mid f_2)\Psi(r_1, r_2)dr_1 dr_2$$

$$\Psi(r_1, r_2) = \begin{cases} 1 & \Pr(f_1 > f_2 \mid r_1, r_2) > \frac{1}{2} \\ 0 & \text{otherwise} \end{cases}$$

where r_1 and r_2 are the (noisy) internal representations of the frequencies of the 1st and 2nd tone.

$$\Pr(f_1 > f_2 \mid r_1, r_2) = \int_{-\infty}^{\infty} \Pr(f'_1 \mid r_1) \int_{-\infty}^{f'_1} \Pr(f'_2 \mid r_2) df'_1 df'_2$$

$$\Pr(f_i \mid r_i) = \frac{\Pr(r_i \mid f_i)\Pr(f_i)}{\int_{-\infty}^{\infty} \Pr(r_i \mid f'_i) df'_i}$$

where $\Pr(f_i)$ is the prior distribution for stimulus i (which is based on the empirical prior distribution), $\Pr(r_i \mid f_i)$ is the probability distribution of neural

responses r_i given the stimulus f_i, which is assumed to be Gaussian with mean 0 and standard deviation σ_i,

$$\Pr(r_i \mid f_i) = \frac{1}{\sqrt{2\pi\sigma_i^2}} e^{-\frac{(r_i - f_i)^2}{2\sigma_i^2}}$$

This model is characterized by two parameters, the standard deviations of the noise σ_1 and σ_2. These parameters were chosen as to minimize the square distance between the vector of choices A and the predictions of the model, $\Pr(\text{"} f_1 > f_2 \text{"} \mid f_1, f_2)$. 65 data-points (trials 16-80 of each block) were used for the fit.
